# Development of the Second Generation Berry Impact Recording Device (BIRD II)

**DOI:** 10.3390/s150203688

**Published:** 2015-02-05

**Authors:** Rui Xu, Changying Li

**Affiliations:** College of Engineering, The University of Georgia, 200 D.W. Brooks Drive, Athens, GA 30602, USA; E-Mail: asilan@uga.edu

**Keywords:** sensing system, blueberry, accelerometer, instrumented sphere, microcontroller

## Abstract

To quantitatively measure the impacts during blueberry harvesting and post-harvest handling, this study designed the second generation Berry Impact Recording Device (BIRD II) sensor with a size of 21 mm in diameter and a weight of 3.9 g, which reduced the size by 17% and the weight by 50% compared to the previous prototype. The sensor was able to measure accelerations up to 346 *g* at a maximum frequency of 2 KHz. Universal Serial Bus (USB) was used to directly connect the sensor with the computer, removing the interface box used previously. LabVIEW-based PC software was designed to configure the sensor, download and process the data. The sensor was calibrated using a centrifuge. The accuracy of the sensor was between −1.76 *g* to 2.17 *g*, and the precision was between 0.21 *g* to 0.81 *g*. Dynamic drop tests showed that BIRD II had smaller variance in measurements than BIRD I. In terms of size and weight, BIRD II is more similar to an average blueberry fruit than BIRD I, which leads to more accurate measurements of the impacts for blueberries.

## 1. Introduction

Blueberries, along with other berry fruits, are perishable and can be easily bruised during harvesting and post-harvest handling, due to a large number of mechanical impacts created by machine parts. It was estimated that about 78% of mechanically harvested blueberries were bruised and unmarketable [1]. Therefore, it is important to fully understand how the blueberry interacts with the machine parts during machine harvesting and handling in order to reduce bruising.

The idea to design a sensing tool to quantitatively measure the mechanical damage during harvesting and post-harvest handling has been put forward by researchers since the early 1970s with the concept of the instrumented sphere. Essentially, an instrumented sphere (IS), also known as the pseudo-fruit, is a standalone data logging device that can measure and record the mechanical impacts experienced by agriculture products. Herold *et al.* defined three technical requirements for an instrumented sphere: (1) the physical properties (e.g., size, mass, shape and elasticity) of the sphere should be similar to the properties of the real object that it intends to measure; (2) it should be able to detect the mechanical damage, including dynamic impacts and static load; and (3) the way that it goes through the practical harvesting and handling processes should represent the way that the real produce does [2]. The second requirement highlights the essential functions of an IS and inspires two different technical directions that have been used by researchers: one direction is to use accelerometers to measure dynamic impacts, and the other direction is to use pressure sensors to measure static load. Some early models of accelerometer-based ISs were capable of recording all impact events above a programmed threshold based on measured acceleration values, but were not able to detect static load [3–5]. Therefore, the second research direction of IS was brought up by Herold *et al.*, who designed a pressure measuring sphere (PMS) and its advanced version, PMS-60 [2]. The PMS-60 can record static load, as well as dynamic impact, but the error (22% variance at a sphere single orientation) is five-times higher than the static load. Another instrumented sphere that can measure dynamic impact using an accelerometer and static load using load cells was developed by Muller *et al.* [6].

Recently, Roa *et al.* developed a wireless instrumented sphere (WIS) that can measure and display impacts in real time through a radio frequency transmitter [7]. Geyer *et al.* developed a miniaturized tri-axial acceleration measuring unit (AMU) that can be embedded into a potato tube, and the AMU recorded average impact loads 1.11-times higher than what were recorded by PMS-60 [8,9]. Some commercialized ISs were also introduced into the market, including the Impact Recording Device (IRD) (Techmark Inc., Lansing, MI, USA), Smart Spud (Most Accurate Sensor Impact Technology, Moncton, Canada), PTR 200 (Potato-shaped Instrumented Device 200) (SM Engineering, Nakskov, Denmark) and TuberLog (ESYS GmbH, Berlin, Germany) [10,11].

However, the previously designed and commercially available ISs were mainly designed for large fruits and vegetables, and none of them can be readily applied to small fruits, like blueberry, due to a significant difference in size and weight. For example, the sizes and shapes of IRD, PMS-60, and PTR 200 are 57-mm sphere, 62-mm sphere and 53 × 53 × 83 mm semi-ellipse, respectively. Their weights are 89 g, 180 g and 170 g, respectively. In contrast, the size of the blueberry is between 7 mm to 23 mm, and the weight is between 0.25 g to 3.91 g [12]. Therefore, developing an IS for blueberries must overcome the challenges of reducing the size and weight of the IS to the range that is comparable to a typical berry fruit. Other challenges include low power consumption with a small-sized battery, a large impact detection range and proper casting material of similar surface properties as a blueberry.

In a previous project, Yu *et al.* designed the first generation of the Berry Impact Recording Device (BIRD) sensing system, consisting of a BIRD I sensor, an interface box and PC software [13,14]. The size of the BIRD sensor is one inch (25.4 mm) in diameter, similar to a large-sized blueberry (23 mm). Its weight (14 g), however, is much greater than that of a normal blueberry. The primary objectives of this study were to develop and characterize the second generation BIRD (BIRD II) sensor with a smaller size, less weight, lower power consumption and lower cost than BIRD I, enabling it to better simulate real berry fruits. Other enhancements of the sensor include removing the interface box by implementing the Universal Serial Bus (USB) in the sensor and improving the sensor and PC software design. The performance of the BIRD II sensor was also characterized and compared with BIRD I.

## 2. Sensor Development

**Figure 1.**
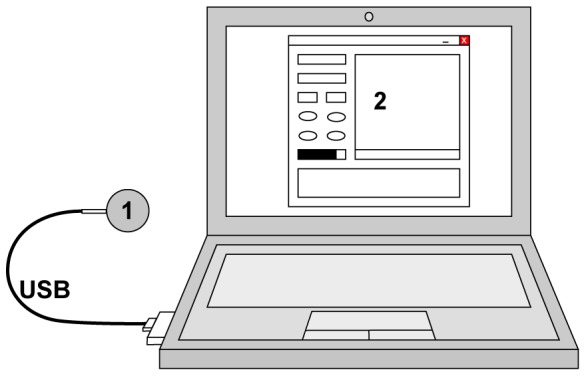
Connection between the Berry Impact Recording Device II (BIRD II) and the PC through a USB cable with a customized connector in the sensor end. The diagram is not drawn to scale. 1, BIRD II; 2, PC and BIRD II PC software.

With a smaller sized circuit board and battery, BIRD II (Figure 1) is downsized to 21 mm in diameter with a weight of 6.9 g, which is reduced by 17% in size and 50% in weight compared to BIRD I. Most parameters of BIRD II are maintained the same as or better than BIRD I (Table 1). The sensing range and maximum sampling frequency of BIRD II are reduced to ±200 *g* (*g* = 9.8 m/s^2^) and 2 KHz compared to BIRD I, but are still technically adequate to record and assemble most impacts occurring in the field. Unlike BIRD I and other ISs, BIRD II can be directly connected to a PC through a Universal Serial Bus (USB) cable without an interface box, which significantly simplifies the operation of the sensor and reduces the possibility of the malfunction of the sensor in the field. Due to these improvements, the total cost of BIRD II is also reduced significantly (by 78%) compared to BIRD I.

### 2.1. Hardware Design

**Figure 2.**
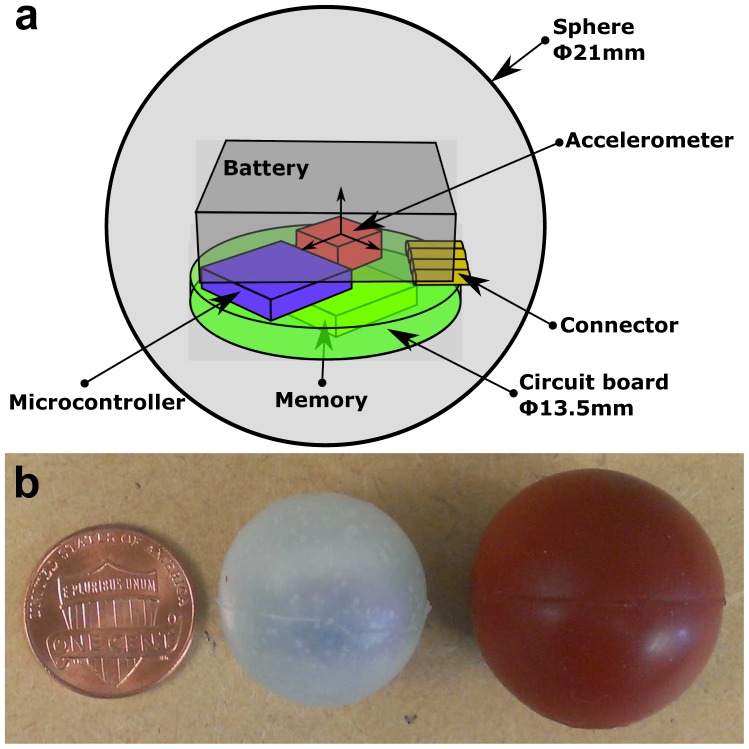
Structure of BIRD II (**a**) and the size comparison of a penny, BIRD II and BIRD I (from left to right) (**b**).

As a recording device, the basic functions of BIRD II are to measure the acceleration, store the data into memory and transfer the data to the computer. Therefore, the sensor should have an accelerometer to measure the acceleration, onboard memory to store the data, a microcontroller to control other units and a battery to power the sensor. To maintain a reasonable data collection performance, the sensor should have enough sampling frequency to avoid aliasing and sufficient measurement range and accuracy to record critical impacts. Due to the capacity of the battery, the selected electronic components should have low power consumption in order to maximize the operation time. Additionally, the selected microcontroller should support the USB protocol in order to connect BIRD II with the PC directly without an interface box. Overall, BIRD II (Figure 2) consists of four essential parts: a tri-axis accelerometer (ADXL377), a 1-Mb ferroelectric random access memory (F-RAM) chip (FM25V10), a microcontroller (PIC18LF14K50) and a rechargeable battery (PGEB201212). Other low-power components were also chosen. The performance of the main components is discussed below.

#### 2.1.1. Accelerometer

In order to reduce the size of the sensor, a trial-axial accelerometer (ADXL377, Analog Device, Norwood, MA, USA) is used in BIRD II. It has ±200 *g* for all sensing ranges in each axis with a typical sensitivity of 6.5 mV/*g*, three times that of BIRD I. Additionally, ADXL377 has low power consumption with a typical 300 μA current drain and a small-sized package (3 × 3 × 1.45 mm), which are both essential criteria for the selection of the accelerometer. The maximum bandwidth of ADXL377 is 1 KHz in each axis, which requires at least a 2-KHz sampling rate.

From the previous tests for blueberry machine harvesters using BIRD I, the results showed that the duration of the impacts on average was 5–10 ms, and the magnitude of most impacts was under 400 *g* [15]. Preliminary packing line tests using BIRD I also showed that most impacts are less than 200 *g*. Therefore, the sensing range and bandwidth of ADXL377 are sufficient to record most impacts without aliasing.

#### 2.1.2. Memory

The choice of the data storage is evaluated from three main criteria: the writing speed of the memory should be fast enough to store the data at a high frequency (up to 2 KHz); the power consumption should be small; and the capacity of the memory should be large enough to store sufficient data points. Other criteria, such as a long life cycle and a small package, are also considered. According to these criteria, a serial F-RAM chip (FM25V10, Ramtron, Colorado Springs, CO, USA) with 1-Mb capacity was used in BIRD II. FM25V10 has a fast Serial Peripheral Interface (SPI) with a 40-MHz maximum clock speed, which provides a sufficient data transfer speed. With an optimized data storage structure, the memory can save up to 16,384 impact data points, which is 82% larger than what BIRD I can store (9000 data points) [14].

#### 2.1.3. Microcontroller

An eight-bit microcontroller (PIC18LF14K50, Microchip, Chandler, AZ, USA) was selected because of its fast operation speed, low power consumption and small package size. PIC18LF14K50 has a 10-bit ADC with nine external channels, a Master Synchronous Serial Port (MSSP) module, which can be used as the SPI, and a Universal Serial Bus (USB) module, which is compliant with the USB V2.0 standard. It has a 16-bit timer, which can be used to generate an accurate system clock for BIRD II. The microcontroller is implemented with nanoWatt eXtreme Low Power (XLP)technology, consuming a small current drain in the active (8 mA) and sleep mode (24 nA). Its program memory and data memory are 16 Kbytes and 768 bytes, respectively, which are sufficient for programming and data manipulating.

#### 2.1.4. Universal Serial Bus

**Figure 3.**
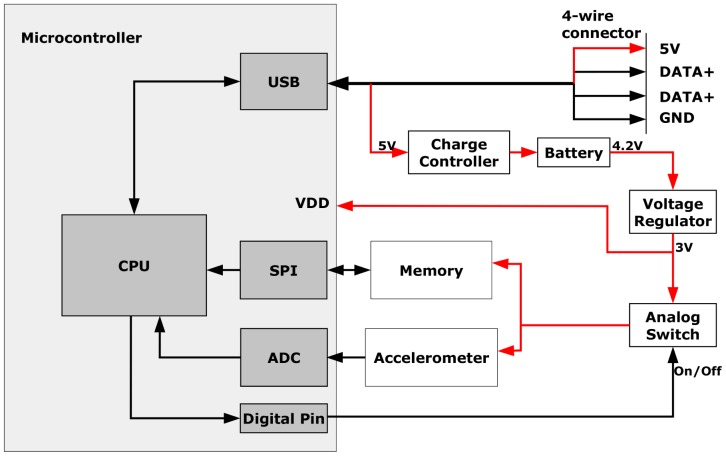
Circuit diagram. The red line indicates the power supply lines of the circuit.

Using the built-in USB transceiver in the microcontroller, BIRD II implemented the USB 2.0 protocol, and only four wires are needed to set up communication with computers. As a result, the interface box is no longer needed for BIRD II, which greatly simplifies the connection between the sensor and computer compared to BIRD I. The communication speed for USB (12 Mbits/s) is also substantially faster than serial communication (115.2 Kbits/s). Consequently, the data downloading time is significantly decreased from 103 s for BIRD I to 8 s for BIRD II. In addition to acting as a communication bridge between the sensor and the computer, the USB also can charge the sensor through the charge controller (Figure 3).

#### 2.1.5. Power Supply

To further reduced the size of the sensor, a lithium-ion rechargeable battery (GM041215-PC, Lund Instrument Engineering, Inc., Orem, UT, USA) with a 3.7-V output voltage and a 45-mAh capacity was selected. The dimensions of the battery are 12 × 10 × 4 mm, and its weigh is 1 g. The battery also comes with a battery protection circuit board that can prevent it from over-discharging and charging. A linear charge management controller (MCP73831, Microchip, Chandler, AZ, USA) was applied to charge the battery through the USB, and the battery can be charged as long as the sensor is connected with the PC. In order to extend the working time of the sensor, low-power chips were used, and the hardware and software were carefully designed to reduce the overall current drain to a desired range. A linear low-dropout (LDO) voltage regulator (MCP1700, Microchip, Chandler, AZ, USA) was employed to regulate the battery voltage to 3.0 V to provide steady power supply for other parts of the circuitry. The microcontroller is directly powered by 3.0 V, while the accelerometer and memory are connected to 3.0 V through an analog switch (ADG752, Analog Device, Norwood, MA, USA), so that the microcontroller can turn them on or off according to different power consumption requirements.

**Figure 4.**
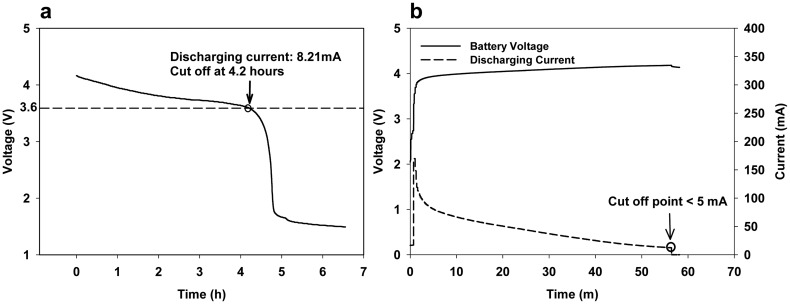
Battery discharging curve (**a**) and charging curve (**b**).

The charging and discharging performance of BIRD II were tested using the following procedure. After fully recharging the sensor and making it sample at the maximum sampling frequency, the voltage of the battery was recorded until the battery was fully discharged. Then, the sensor was charged by the computer via a USB cable, and the voltage of the battery and the recharging current was recorded until it was fully charged (the recharging current became zero). The discharging curve (Figure 4a) shows that the battery reached the cutoff voltage (3.6 V) at 4.2 h with an 8.21-mA discharging current, indicating that the sensor can work at least four hours. The charging curve (Figure 4b) shows that the battery can be fully charged in less than an hour.

#### 2.1.6. Housing Design

The circuit board and battery were directly cast into a 21-mm (in diameter) sphere using silicon rubber (AM 128T, AeroMarine Products Inc., San Diego, CA, USA) (Figure 2a). According to the manufacture's data sheet, the tensile strength at 400% elongation of the silicon rubber is 3.96 MPa, which is equivalent to a Young's modulus of 0.79 MPa. For comparison, the Young's modulus of Maine wild blueberries is between 0.014 MPa and 0.026 MPa [16]. The final size of BIRD II was reduced by 17% compared to BIRD I. The weight of the sensor is 6.9 g, which is reduced by 50% compared to BIRD I. The weight distribution of the BIRD I and BIRD II is listed in Table 2, which shows that the weight reduction is mainly contributed to by the reduction of the battery and the housing material.

### 2.2. Software Design

#### 2.2.1. Sensor Program

**Figure 5.**
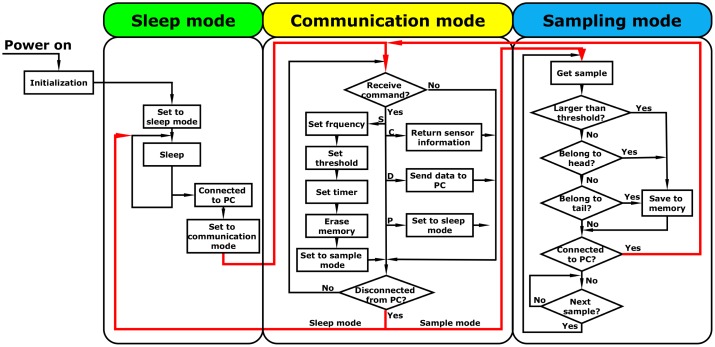
Flowchart of the sensor program. “S”, configure sensor and set sensor to sampling mode; “C”, request sensor information; “D”, upload sensor data to the PC; “P”, set sensor to sleep mode. These letters indicate different types of commands.

The sensor can operate at three different modes (Figure 5)—sleep, communication and sampling mode—based on the different power consumption levels and functions of the sensor. Once the sensor is powered on, it makes the necessary initialization for the hardware and program, then it goes to sleep mode by default. Under sleep mode, the accelerometer and memory are powered off, and the microcontroller is suspended and only responds to an external interrupt. Therefore, the power consumption of the sensor will be maintained at the lowest level with a current drain of 0.01 mA. The sensor will change to communication mode after being connected to the PC to receive and process commands from the PC. After receiving a command, the sensor executes the command according to the command type (indicated by different letters). For example, if the sensor receives the “start to sample” command, the sensor will set the frequency, threshold and system time based on the user's setting in the PC program, then erase the sensor memory and set the sensor to sampling mode. The sensor starts to sample only after being disconnected from the PC.

In the sampling mode, the sensor measures the acceleration and stores the results into the memory at a sufficient frequency. A typical impact is in a bell curve shape. A user-defined threshold is used to avoid recording trivial impacts that are below the threshold. To make the impact curve complete, certain data points below the threshold are also recorded: seven before and seven after the impact curve, defined as leaders and trailers, respectively. In each sampling cycle, the sensor first converts the analog acceleration voltage into a digital value and then compares this with the threshold using the summation acceleration, which is defined as the scalar value of the vector summation of the three axes [13]. If the summation acceleration is greater than the threshold, the acceleration value, as well as the leaders and trailers of that impact will be written to the memory.

#### 2.2.2. “pBIRD” PC Program

**Figure 6.**
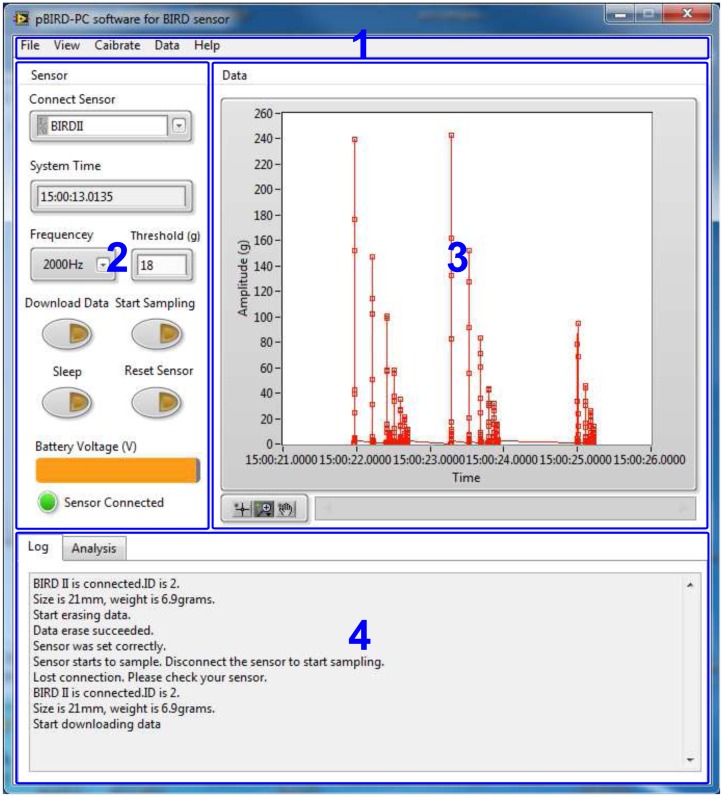
Front panel of the main PC program. **1**, Menu bar; **2**, sensor configuration; **3**, data visualization; **4**, operation history.

**Figure 7.**
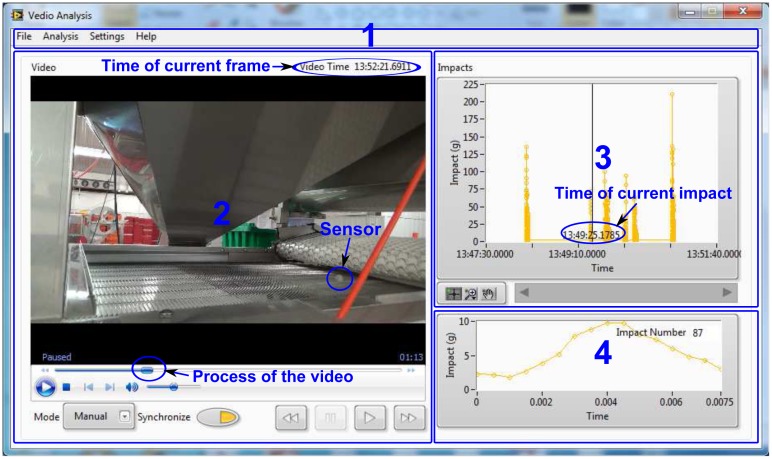
Front panel of the video analysis sub-program. 1, Menu bar; 2, video display and control; 3, overall impacts display; 4, single impact display The video and impact data come from a field test on a blueberry packing line. It clearly shows that the impacts near the cursor occurred when the sensor was transferred from the white conveyor belt to the steel conveyor belt. The program uses relative time to synchronize the display between the video and impacts, and even their absolute time is different.

In order to manage the sensor, a PC program “pBIRD” was designed using LabVIEW 2013 (National Instruments, Austin, TX, USA). The PC program has three main functions: sensor configuration, data processing and video analysis. These functions are accomplished by a main program (Figure 6) and a sub-program (Figure 7).

The program can plot the velocity change (VC) *versus* peakGand the time sequence of the summation acceleration and the acceleration of each single axis. Before plotting the data on the program, the raw data need to be converted to real acceleration. The conversion of each axis is achieved based on Equation (1).

(1) Acceleration(g)=(D−O)×0.45g

where D is the output of the AD conversion, O is the output of the AD conversion when the measured axis is under zero acceleration (placed horizontally) and 0.45 *g* is a coefficient that was determined by the resolution and the reference voltage of the AD converter and the resolution of the accelerometer, which is given by the manufacture. The summation acceleration was the scalar of the acceleration vector, which is calculated through Equation (2). Therefore, the sensor can measure summation acceleration up to 346 *g*.

(2) $$\text{Summation}=\sqrt{{\text{X}}^{2}+{\text{Y}}^{2}+{\text{Z}}^{2}}$$

After converting the raw data to acceleration, the “velocity change (VC)” and “peakG” can be derived from the summation acceleration. The “velocity change (VC)” of one impact is defined as the area under the impact curve, which is calculated by integrating the summation acceleration over time. The “peakG” of one impact is defined as the largest summation acceleration. The VC *versus* peakG and the time sequence of the summation acceleration and the acceleration of each single axis can be plotted based on the user's selection.

The video analysis subprogram (Figure 7) consists of three parts: a video player that can display and control the video; an overall impact plot to show all of the impacts in one plot; and a single impact plot to display the details of the current impact. The cursor in the overall impact plot is used to indicate the current impact and can move according to the display of the video. The subprogram uses relative time to link the video and the impact data, so the time of the video camera and of the sensor does not necessarily need to be synchronized in advance. To get the relative time, the user first needs to specify a reference time for the video and impact data by selecting the absolute times when the first impact appears in the video and in the impact data, which can be achieved by dragging the cursor or the video process bar to the desired place. The program then calculates the relative time of the current video frame to the reference time. Finally, the absolute time of the cursor in the overall impact plot is updated by adding the relative time of the video to the absolute time of the time reference for the impact data.

## 3. Calibration and Characterization

### 3.1. Sensor Calibration

**Figure 8.**
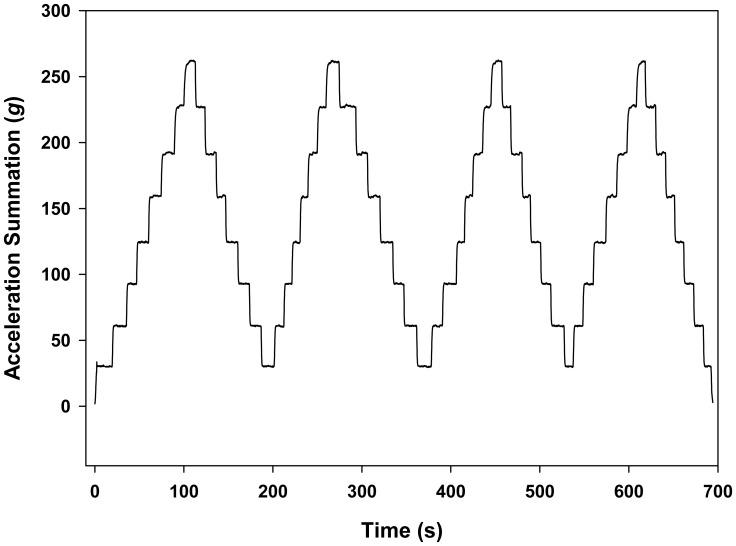
Raw acceleration recorded by BIRD II during calibration.

In order to get a higher range of reference acceleration, the sensor was calibrated using a centrifuge (Centrifuge 5430R, Eppendorf, Hamburg, Germany) based on the procedure described by Yu *et al.* [13]. Eight rotational speeds (690, 970, 1190, 1370, 1540, 1680, 1820 and 1940 RPM) were used to create eight acceleration values of 25.21 *g*, 49.82 *g*, 74.98 *g*, 99.37 *g*, 125.57 *g*, 149.43 *g*, 175.38 *g* and 199.27 *g*. The speed of the centrifuge was changed from the lowest to the highest rotational speed. The sensor was set to sample with a threshold of 18 *g* at a sampling frequency of 10 Hz to avoid memory overflow during the calibration. Each rotational speed was kept for several seconds to allow the sensor to record enough samples, and 40 samples were used as one replicate. The mean value of each replicate was used as the measured acceleration. After the centrifuge reached a 1,940-RPM rotation speed, the centrifuge was slowed down to the lowest rotational speed. This up and down cycle was repeated four times (Figure 8). In total, there are five replicates for 690 RPM, four replicates for 1940 RPM and eight replicates for 970, 1190, 1370, 1540, 1680 and 1820 RPM. A linear regression analysis between the reference acceleration and the measured accelerations was performed using MATLAB R2013b (MathWorks, Inc., Natick, MA, USA), and the best-fit line was used to calibrate the sensor. After calibration, the precision and accuracy of the sensor were evaluated by the standard deviation of each replicate and the deviation of the measured acceleration after calibration from the reference acceleration.

### 3.2. Surface Property Test

**Figure 9.**
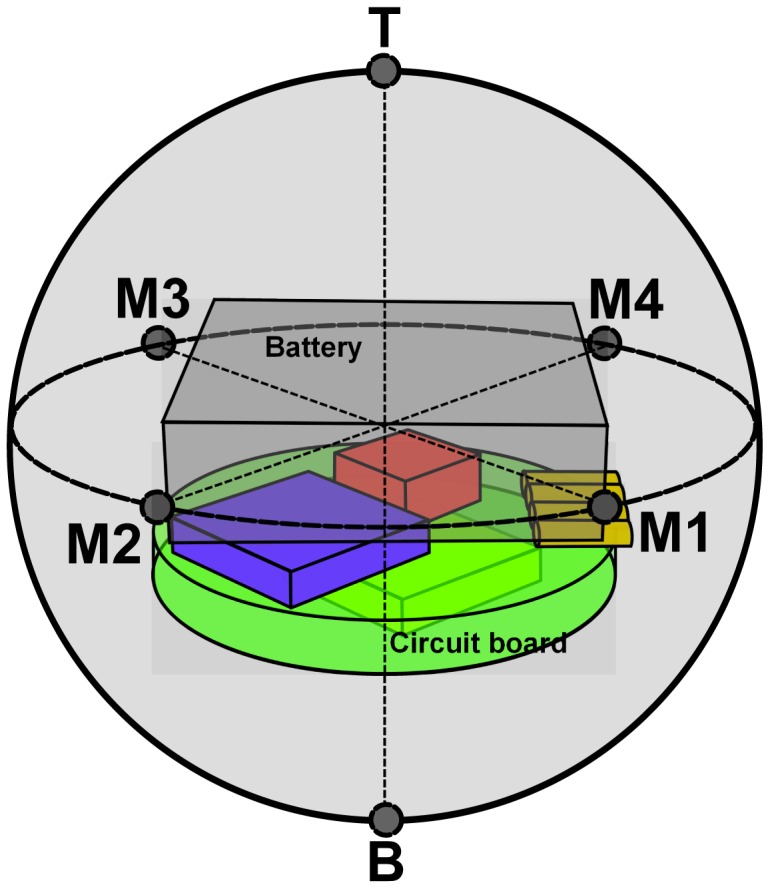
Schematic of the location of the six measured points.

To compare the surface properties of the blueberry and BIRD sensors, the compression force at 1-mm deformation was measured for both BIRD I and BIRD II using a texture analyzer (TA XT2i, Stable Micro Systems LTD., Godalming, UK) and compared with the data from the blueberry fruit. BIRD I and BIRD II were fixed on the base of the texture analyzer using a spherical sample holder. A cylinder probe was used to compress the two sensors by 1 mm, and the compression force was measured. The average value was used by measuring six different locations, namely four points at the quartiles of the equator (respectively, M1, M2, M3 and M4) and two points at two poles (Tand B) (Figure 9).

### 3.3. Sensor Dynamic Drop Test

In order to evaluate the performance of BIRD II and to compare it with BIRD I, dynamic drop tests of both BIRD I and BIRD II were conducted. Both of the sensors were dropped from five drop heights of 2.5, 5, 7.5, 10 and 12.5 cm with 40 replicates for each drop height. A 1.2-cm padding sheet (Poron “No-Bruise”, A & B Packing Equipment Inc., Lawrence, MI, USA) and a 0.2-cm steel sheet were selected as the contact materials for their different surface properties. The steel is a hard material (higher elastic moduli) with little energy absorption and commonly used in machines, while the padding sheet has a strong cushioning effect that can absorb more energy. The mean and standard deviation of the peakG and VC are used in the data analysis. Although there were several impacts (bounces) at each drop, only the first impact of each drop was considered. Theoretically, the same sensor should record different responses on different contact materials, and BIRD I and BIRD II should have different responses for a given contact material due to the different housing materials.

### 3.4. Sensor Surface Uniformity Test

The variance of the IS measurements is mainly contributed by two sources: the precision of the sensing unit (e.g., accelerometer, pressure sensor) and the inhomogeneity of the IS. Essentially, any inconsistency (e.g., uneven thickness, flat spot and irregular shape) in the IS will affect the reading [17]. For example, the pressure measured by the pressure measuring sphere (PMS60) spreads by 13.8% at different orientations given the same static pressure [2]. The potato-shaped instrumented device (PTR 200) also measures significantly different values between different impact zones given the same impact force under the pendulum test [18].

**Figure 10.**
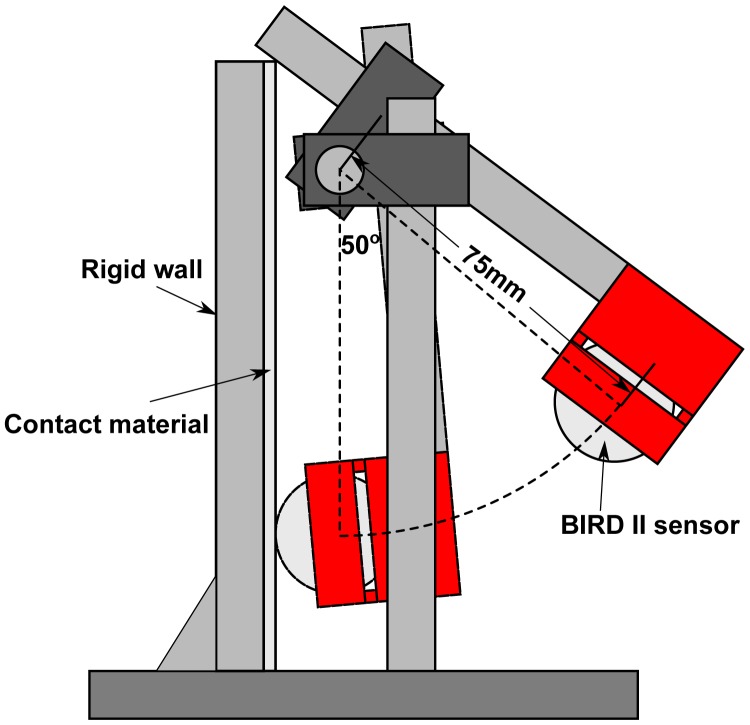
Schematic of the pendulum test (side view).

To have an overall view of how BIRD II responds at different collision points, a uniformity test was performed using a pendulum. BIRD II was attached to one end of the pendulum (Figure 10) to fix the collision point and was released to collide with the contact material against a rigid vertical wall. The same six points used in the sensor surface property test (Figure 9) were chosen as collision points. The same contact materials (padding sheet and steel) used in the dynamic drop test were tested. Each collision point was tested 30 times, and only the first impact of each drop was used for analysis. An ANOVA test was performed on the peakG value to test whether there were statistic differences between the impact values from the six points.

## 4. Results and Discussion

### 4.1. Sensor Calibration

**Figure 11.**
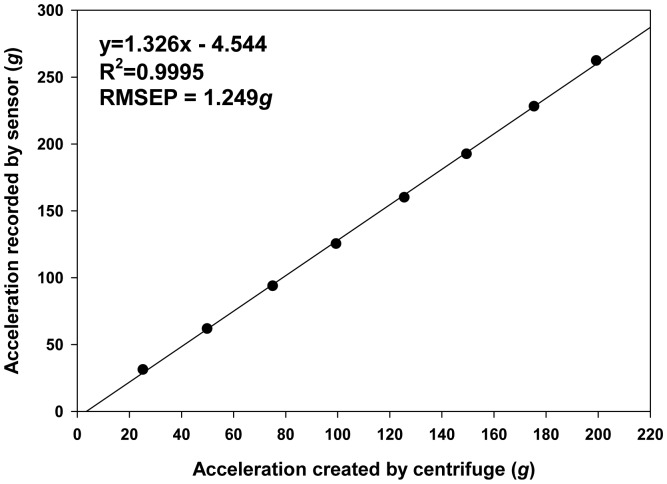
Linear relationship between the acceleration provide by the centrifuge and the values recorded by BIRD II.

**Figure 12.**
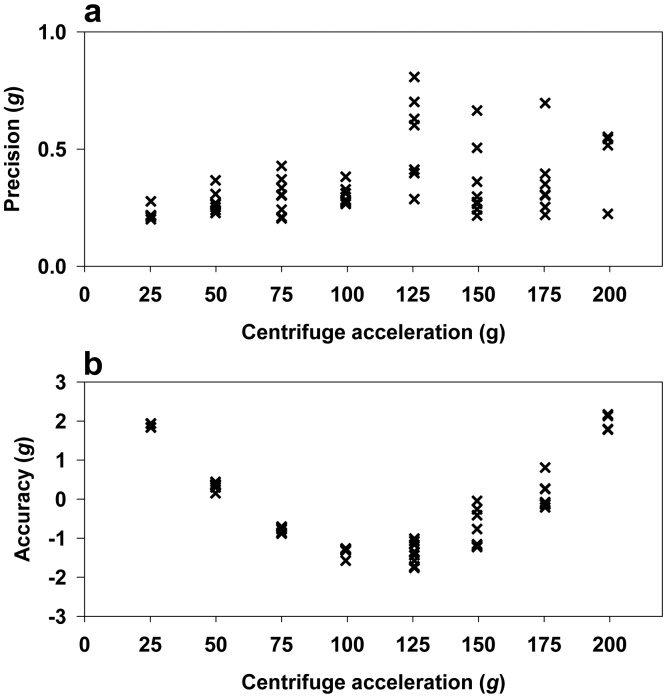
BIRD II calibration: precision (**a**) and accuracy (**b**) of BIRD II.

A strong linear relationship with a coefficient of determination (*R*^2^) of 0.99 and a root mean square error of prediction (RMSEP) of 1.249 g was observed between the reference acceleration and the measured acceleration (Figure 11). Figure 12a showed that BIRD II has better precision (0.20 *g* to 0.81 *g*) than BIRD I (0.32 *g* to 3.11 *g*) [13]. The increase of precision in BIRD II is largely because of the increase of the sensitivity of the accelerometer due to the reduction in the sensing range. The accuracy of BIRD II (Figure 12b) is limited from −1.76 *g* to 2.17 *g*, which is similar to the accuracy of BIRD I (−1.62 *g* to 2.60 *g*) [13].

### 4.2. Sensor Surface Property Test

Table 3 shows that a smaller force was measured for BIRD II (5.54 N) than BIRD I (26.56 N), indicating that the surface property of BIRD II is softer than BIRD I. Compared to the results measured by Saftner *et al.* (2008) for blueberries of multiple cultivars under the same condition, both BIRD I and BIRD II showed higher values (or higher firmness) than blueberry fruit (1.37–1.86 N) [19]. Therefore, the BIRD sensors may record a higher impact than what a blueberry may experience. It is also noteworthy that the compression force at different test points for both BIRD sensors are different, indicating that the BIRD sensors are not homogeneous, which can be a factor that contributes to the variance of the sensor measurements, as discussed in the next section.

### 4.3. Sensor Dynamic Drop Test

**Figure 13.**
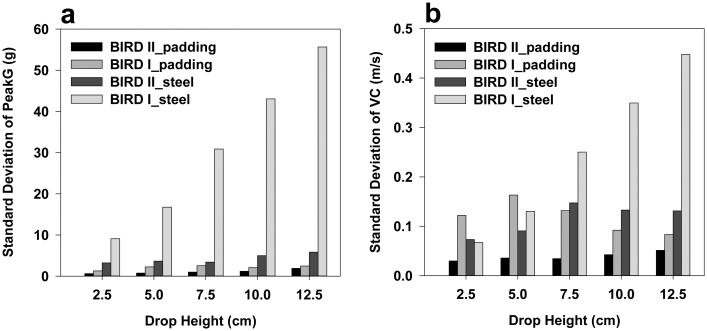
Variance of the dynamic drop measurements by BIRD I and BIRD II. Standard deviation of peakG (**a**) and standard deviation of velocity change (VC) **(b)**.

The standard deviations of the peakG (Figure 13a) measured by BIRD II were smaller than those measured by BIRD I for all drop heights on both the steel and padding sheet, although the standard deviation varied over different drop heights. For BIRD II, the peakG measured on the padding sheet and steel surface had standard deviations ranging from 0.64 *g* to 1.91 *g* and from 3.52 *g* to 5.95 *g*, respectively. For BIRD I, the peakG measured on the padding sheet and steel surface had standard deviations ranging from 1.31 *g* to 2.58 *g* and from 9.11 *g* to 55.68 *g*, respectively. BIRD II had a smaller standard deviation in VC than BIRD I for all drop heights on the padding sheet and for all drop heights, except 2.5 cm, on the steel surface (Figure 13b). Overall, BIRD II had smaller variance in measuring dynamic impacts than BIRD I.

For both the sensors, the standard deviations of the peakG and VC measured on the padding sheet were smaller than on the steel surface for all of the heights. A possible reason could be that the cushioning provided by the padding sheet reduced the difference caused by the inhomogeneity of the sensor, while the steel tended to increase the difference due to its hard surface.

**Figure 14.**
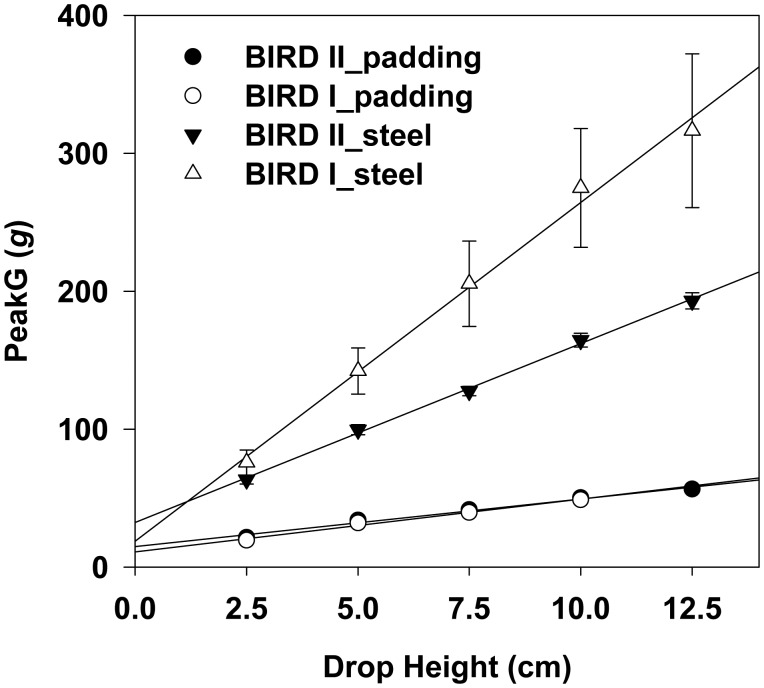
Mean peakG measured by BIRD I and II at different drop heights on two contacting materials.

For the same drop height, the mean peakG values measured by BIRD II on the steel surface were smaller than those measured by BIRD I (Figure 14). No significant difference was found on the padding sheet, which could be caused by the fact that the cushioning effect of the padding sheet reduced the response difference between BIRD I and BIRD II. Both of the sensors recorded higher mean peakG on the steel than on the padding sheet, which was consistent with the texture of the contact material. Linear relationships between the mean peakG and drop height were observed for BIRD I and BIRD II with coefficient of determinations (*R*^2^) of 0.989 and 0.985 for the steel surface, and of 0.994 and 0.998 for the padding sheet, respectively.

### 4.4. Sensor Surface Uniformity Test

**Figure 15.**
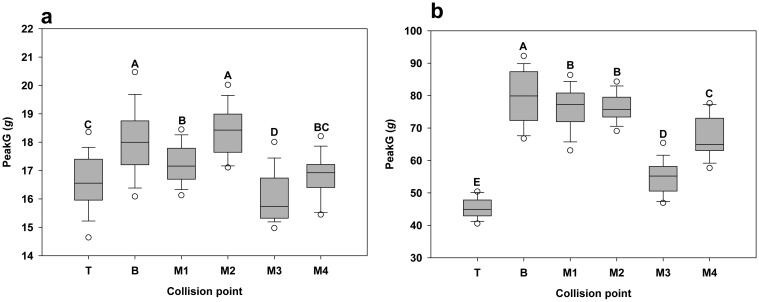
Uniformity test results for padding sheet (**a**) and steel (**b**). The collision points with the same letter indicate that there was no significant difference between them.

As shown in Figure 15, the sensor recorded different peakG values at different collision points. An ANOVA test showed that there was a significant difference (*p*-value < 0.0001) between different collision points. The different responses of the sensor over different collision points contribute to the variance of the measurements under the dynamic impact. It was also observed that the variance among the six collision points on the padding surface (3.144 *g*) was less than on the the steel surface (44.995 *g*), suggesting that the mechanical properties of the contact surface also affect the variance of the sensor measurement.

The variation of the six collision points of BIRD II could be caused by several factors, such as the shape of the housing mold not being perfectly spherical, the accelerometer, circuit board and battery not being positioned in the center and air bubbles in the housing material. These factors are introduced in the housing process and are usually sensor dependent. It is a challenge to make the sensor surface uniform, mainly because it is difficult to keep the thickness of the cast material around the sphere uniform due to the limitation of the small size of the sensor. To address this issue, it is necessary to have adequate replicates to minimize the uncertainty of the measured mean value when the BIRD sensor is used in the field.

## 5. Conclusions

Compared to BIRD I, BIRD II is 17% smaller in diameter and 51% lighter in weight. The performance of BIRD II consistently surpasses that of BIRD I in terms of sensitivity, precision, memory capacity, cost, and ease of use. BIRD II gives a more accurate approximation to a berry fruit compared to BIRD I. However, challenges still remain in making the surface texture property of the BIRD sensor more similar to fruit texture, as well as in improving the surface uniformity of the sensor.

Although BIRD I and II are primarily designed for small fruits and vegetables in studying their interactions with machine harvesters and packing lines, they can be used for other small fruits and vegetables. They also can be used for large fruits and vegetables with moderate modification, for example embedding the sensor into the produce. The sensor also offers the opportunity for blueberry growers to evaluate the design of their packing lines and reduce the potential damage to the produce.

## Figures and Tables

**Table 1.** Comparison between BIRD I and BIRD II.

**Parameter**	**BIRD I**	**BIRD II**
Size	Φ25.4 mm	Φ21 mm
Weigh	14 g	6.9 g
Sensing range	±500 *g* for each axis	±200 *g* for each axis
Sensitivity *^a^*	2.2 mV/g	6.5 mV/g
Sampling frequency	Up to 3 KHz	Up to 2 KHz
Memory	Up to 9000 datasets	Up to 16,384 datasets
Operation time	4 h	4 h
Connectivity	Serial communication through an interface box	USB 2.0 with customized connector
Material cost *^b^*	$356.89	$79.68

a *g* = 9.8 m/s^2^.

b Cost includes the interface box for BIRD I.

**Table 2.** Weight distribution of BIRD I and BIRD II (g).

**Sensor**	**BIRD I**	**BIRD II**
Circuit board	1.9	1.2
Battery	3.3	1.2
Housing	8.8	4.5
Total	14	6.9

**Table 3.** Compression force (N) required for 1-mm deformation of BIRD I and BIRD II at six measured points.

**Sensor**	**T**	**B**	**M1**	**M2**	**M3**	**M4**	**Mean**	**SD.**
BIRD I	17.81	30.43	30.66	25.30	23.93	31.21	26.56	4.81
BIRD II	4.87	5.31	6.60	5.17	5.74	5.52	5.54	0.55
